# The Affective Neuroscience of Sexuality: Development of a LUST Scale

**DOI:** 10.3389/fnhum.2022.853706

**Published:** 2022-02-28

**Authors:** Jürgen Fuchshuber, Emanuel Jauk, Michaela Hiebler-Ragger, Human Friedrich Unterrainer

**Affiliations:** ^1^Center for Integrative Addiction Research, Grüner Kreis Society, Vienna, Austria; ^2^Department of Philosophy, University of Vienna, Vienna, Austria; ^3^University Clinic for Psychiatry and Psychotherapeutic Medicine, Medical University of Graz, Graz, Austria; ^4^Department of Psychology, University of Graz, Graz, Austria; ^5^Clinical Psychology and Behavioral Neuroscience, Technische Universität Dresden, Dresden, Germany; ^6^University Department of Medical Psychology and Psychotherapy, Medical University Graz, Graz, Austria; ^7^Department of Religious Studies, University of Vienna, Vienna, Austria

**Keywords:** LUST, questionnaire development, factor analysis, primary emotions, Affective Neuroscience

## Abstract

**Background:**

In recent years, there have been many studies using the Affective Neuroscience Personality Scales (ANPS) to investigate individual differences in primary emotion traits. However, in contrast to other primary emotion traits proposed by Jaak Panksepp and colleagues, there is a considerable lack of research on the LUST (L) dimension – defined as an individual’s capacity to attain sexual desire and satisfaction – a circumstance mainly caused by its exclusion from the ANPS. Therefore, this study aims to take a first step toward the development of a standardized self-rate measurement for the L-disposition. For this purpose, two versions of the L-scales (L-12 and L-5) were developed and evaluated regarding reliability and aspects of validity.

**Materials and Methods:**

After a pilot study (*N* = 204; female: 81%) with an initial 20-item pool item reductions were conducted. This led to the construction of a 12-item (L-12) version and a 5-item version (L-5), which were assessed in a second sample consisting of 371 German-speaking healthy adults (58.50% female) aged 18–69 years (*M* = 28; *SD* = 9.75). Aspects of external validity were assessed by investigation of correlations with the ANPS, psychiatric symptoms (Brief Symptom Inventory-18), attachment security (Adult Attachment Scales) and personality functioning (Operationalized Psychodynamic Diagnostics Structure Questionnaire). To evaluate structural validity, both L-scales were investigated via confirmatory factor analysis (CFA).

**Results:**

Cronbach’s α indicated excellent internal consistency regarding L-12 (α = 0.90), while L-5 showed acceptable reliability (α = 0.82). CFA of a bifactor model of the L-12 indicated excellent model fit. Moreover, an excellent model fit was observed regarding a single factor model of L-5. For both scales small to moderate positive correlations were observed with SEEKING, PLAY, and secure attachment, while they exhibited small to moderate negative correlations with SADNESS, insecure attachment, lower personality functioning, and increased psychiatric symptom load.

**Conclusion:**

Both newly developed scales exhibit satisfying psychometric properties, indicating high reliability, good structural validity and plausible correlations with external criteria. Hence, this study poses an important step toward the operationalization of the LUST concept. However, more research is needed in particular with respect to the scale’s external validity and its applicability in clinical populations.

## Introduction

Affects are considered as the paramount motivational foundation of human behavior, contributing a significant proportion to the formation of the individuals’ temperament and personality (Kernberg, 2015). In recent decades, several attempts were made to differentiate and categorize basic affective systems (e.g., Izard, 1992; Panksepp, 1998; Ekman, 1999; Krause, 2012). In contrast to more cognitively oriented emotion/affect models, Panksepp (1998) emphasized the role of subcortical brain areas in the emergence of primary emotions [in Affective Neuroscience (AN) theory, *affect* is usually used synonymous with the term *emotion*]. Based on ethological research, he distinguished seven primary emotion networks, neuroanatomically located between the Periaqueductal Gray (PAG) and the limbic forebrain (Panksepp, 1998; Panksepp and Biven, 2012). These cross-species primary-process systems are assumed to act as prototype emotional states, which can be evoked via artificially induced stimulation (Panksepp and Watt, 2011). The Affective Neuroscience Personality Scales (ANPS; Davis et al., 2003) aim to measure six dimensions of these primary emotion systems including SEEKING, ANGER, FEAR, PLAY, SADNESS, CARE, and asses an additional spirituality scale.

While also conceptualized as a primary emotion, LUST was excluded from operationalization in the ANPS as the authors assumed that it “seems less relevant to current conceptualizations of human personality and we also suspected that it may potentially be an affective factor that people would not wish to be frank about, and thus may contaminate frankness on the other scales” (Davis and Panksepp, 2011, p. 1949). Nevertheless, LUST – defined as the “systems that contribute distinctly to female and male sexuality and associated erotic feelings” (Panksepp, 2004, p. 52) – remains a pivotal part of AN and related neuropsychoanalytic theory, involved in important aspects of etiological models concerning psychiatric disorders (Panksepp, 2004; Boeker et al., 2018). Accordingly, it appears sensible to construct a standardized measurement of this dimension in order to facilitate psychometrically oriented neuropsychoanalytic research. This demand is further highlighted by recent progress in this field achieved via the usage of the ANPS concerning investigations of primary emotions and its relationship to personality and phenomena of clinical psychology (Marengo et al., 2021; Montag et al., 2021).

Yet, to the best of our knowledge, no publicly available LUST scale exists until this day. One of the few trackable references to the LUST concept in relation to the ANPS can be found in a conference paper by Lotter and Dauphin (2014), which investigated the relationship between the ANPS and the Sexual Desire Inventory-2 (SDI-2; Spector et al., 1996), indicating a positive correlation between dyadic sexual desire and SEEK and PLAY as well as solitary and total sexual desire with SEEK and ANGER.

van der Westhuizen and Solms (2015) describe the development of a LUST measurement in context with the investigation of social dominance. According to the authors, the scale exhibited good internal consistency and was conceptualized via the assessment of “sexual desire, physiological arousal and sexual mentation.” However, this LUST scale was not made publicly available. Nevertheless, the authors reported moderate positive correlations between LUST and SEEKING and PLAY, small positive correlation with CARE and DOMINANCE as well as a small negative correlation with FEAR.

Another attempt to measure LUST was made by Dolce et al. (2020), who investigated a primary emotion driven personality concept by the means of neuronal networks training (Dolce et al., 2020). While the conceptual strategy of their newly developed self-rate measurement differs rather drastically from the original ANPS, the questionnaire primarily aims to achieve a high predictive validity with respect to psychiatric disorders. Therefore, in its current form, LUST is inversely assessed by three items which focus on inappetence, sadness and loneliness in social situations.

Based on its original definition, the LUST system is linked to feelings of pleasure, sexual urges and gratification, and is reciprocally connected to the SEEKING network (Panksepp, 1998; Solms and Turnbull, 2002; Panksepp and Biven, 2012). Its activation diminishes SEEKING driven appetence behavior and triggers feelings of satisfaction, serving as reward mediating substrate necessary for learning (Solms and Turnbull, 2002). This dichotomy between SEEKING and LUST largely corresponds to Robinson and Berridge’s (2000) differentiation between “wanting” and “liking.” In correspondence to this, the process of learning is understood as a coupling between psychomotor SEEKING impulses (“wanting”) and the experience of pleasure mediated by the LUST system (“liking”).

**FIGURE 1:**
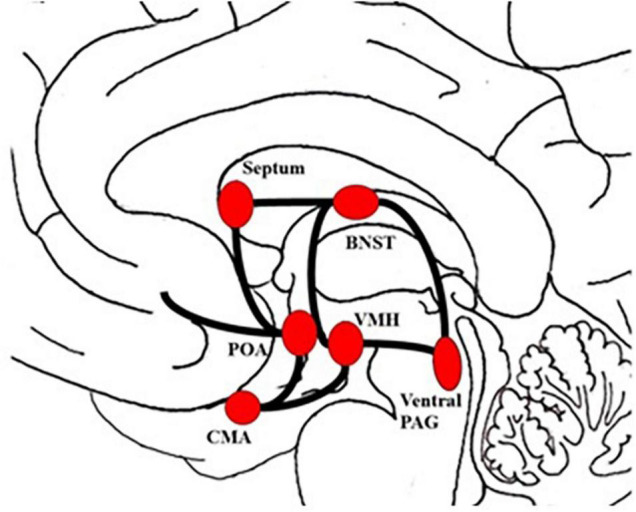
Schematic visualization of the LUST system. The figure was drawn by JF, based on theoretical concepts by Solms and Turnbull (2002) and Panksepp (2011). BNST, bed nucleus of the stria terminalis; CMA, cortico-medial amygdala; POA, preoptic area; PAG, Periaqueductal gray; VMH, ventromedial hypothalamus.

Inferred from animal model, the LUST network is assumed to consist of a complex group of structures, descending from the hypothalamus to the posterior parts of the midbrain (see Figure 1; Panksepp, 1998; Solms and Turnbull, 2002). Most authors agree that the LUST system is composed of the Bed nucleus of the stria terminalis, the central tegmental field, the preoptic area and the ventromedial hypothalamus, the Nucleus accumbens shell, septum area and the ventral Periaqueductal gray (Solms and Turnbull, 2002; Holstege and Huynh, 2011; Panksepp and Biven, 2012; Berridge and Kringelbach, 2015; Hashikawa et al., 2016). Neurochemically, the LUST system is largely controlled by endorphins acting on mu, delta- and kappa-opioid receptors in the Nucleus accumbens shell, and hormones like vasopressin, testosterone and oxytocin (Solms and Turnbull, 2011; Panksepp and Biven, 2012; Berridge and Kringelbach, 2015).

Moreover, recent studies in humans investigating the neural effects of orgasms with PET scans (Georgiadis et al., 2006; Holstege and Huynh, 2011) observed increased blood flow within the upper brainstem and cerebellum. Men showed increased activation in the insula, while women exhibited increased activation in the somatomotor and somatosensory cortex and a deactivation in the left temporal lobe and ventral prefrontal cortex.

Regarding the clinical significance of the LUST system, Panksepp (2004) exclusively proposed associations with disorders directly related to sexuality like sexual dysfunctions, sexual addictions, fetishes, and excessive jealousy. However, with regard to empirical research it seems plausible that dysfunctions of the LUST-system might be involved in a broad range of psychopathologies like affective disorders (Rokach, 2019), addictions (Kumsar et al., 2016), personality disorders (Collazzoni et al., 2017) and schizophrenia (Kelly and Conley, 2004). Yet, while there is growing evidence regarding associations between most primary emotions dispositions and psychopathology (Montag et al., 2021), due to the current lack of appropriate research instruments very little is known about the role of LUST.

As for associations with other personality traits, the current state of psychodynamic literature suggests associations between increased attachment security and personality functioning with a predominance of positive affective traits (Arbeitskreis OPD-2, 2008; Krause, 2012), an assumption supported by recent empirical evidence (Fuchshuber et al., 2019). Thus, it seems plausible to assume that a measure of LUST is positively correlated with increased personality functioning and attachment security. However, with research investigating the role of attachment security in connection to the related concept of erotophilia [defined as positive respond and attitude bias toward sexual cues (Fisher et al., 1988)], indicating no significant correlations, this relationship seems less clear (Bogaert and Sadava, 2002). Despite conceptual overlaps between both erotophilia and a disposition toward LUST, the transferability of this result remains uncertain, as erotophilia might be more oriented toward conscious attitudes regarding sexuality than the personality trait LUST, defined by the individual’s ability to attain sexual desire and pleasure.

### Study Aims

This study aims to develop and psychometrically evaluate a preliminary measurement tool, which enables the assessment of the LUST concept decisively developed and described by Panksepp (1998). Hence, the scale might be seen as an exploratory addition to the already existing ANPS which – at its present form – do not operationalize LUST. In correspondence to this, we examine the reliability as well as aspects of the validity of this newly developed self-rate measurement.

Thereby, we expected the L-scales to correlate positively with adaptive attachment dimensions (trust and closeness) and negatively with attachment anxiety, as well as positively with personality functioning, and negatively with psychiatric symptom load.

## Materials and Methods

### Item Generation

After an extensive literature search on available discourse on LUST as well as existing measurements of sexuality, the authors (JF, EJ, and MH-R) developed an item pool of 20 questions, which aimed to operationalize the concept as outlined by Panksepp (1998), Solms and Turnbull (2002), and Panksepp and Biven (2012). Hence, the scale was conceptualized as a single factor measurement of the individual’s capacity to attain sexual desire, enjoyment and pleasure. Emphasis was placed on comprehensibility of the items, subsequently double negations, foreign words or technical terms were avoided. Furthermore, items were formulated as short and unambiguous as possible (Bühner, 2011).

Half of the items were constructed as negatively scored items and formatted in line with the BANPS (Barrett et al., 2013). Therefore, a five-point Likert scale was used to assess the individual items, as a response format of five to seven levels is suggested to maximize the reliability of assessment tools (Bühner, 2011). “1 = strongly disagree, 2 = disagree, 3 = neither agree nor disagree, 4 = agree, 5 = strongly agree” was chosen as the response format.

### Item Reduction

The initial item pool was tested in a pilot study (*N* = 204; female: 81%). This initial version of the LUST-scale was estimated to have a Cronbach’s α of 0.70. To achieve a reliable, unidimensional and valid scale this version was trimmed by the examination of (1) item-total correlations, aiming to achieve a Cronbach’s α > 0.90 for the long and a Cronbach’s α > 0.80 for the short version, (2) Item difficulty, thereby it was aimed to achieve a mixed set of item difficulties with a predominance of moderately difficult items, (3) Exploratory factor analysis to test for one-dimensionality and (4) considerations regarding construct validity. The trimming steps were carried out iteratively and led to the construction of a 12-item version (L-12). Moreover, by further reducing the number of items a five-item version (L-5) was developed.

### Sample and Procedure

The participants (*N* = 371; 58.50% female) were recruited through advertising on social networks and public announcements at the Karl-Franzens University of Graz and Medical University of Graz. Informed consent was acquired before each participant filled in the test form that included demographic questions (e.g., age, sex, education status, and occupation status), the newly developed LUST scale as well as standardized questionnaires described below. No recompense was provided. The data were acquired via the online-survey platform LimeSurvey. Participants were included if they were aged over 18, stated no history of psychiatric disorders and completed all questionnaires.

The study was carried out in accordance with the Declaration of Helsinki. Ethical approval was granted by the Ethics Committee of the Medical University of Graz, Austria.

### Psychometric Assessment

The German version of the *Affective Neuroscience Personality Scales* [ANPS; Davis et al., 2003; German version by Reuter and Henning (2015)] is a self-report measure which assesses behavioral traits associated with Panksepp’s (1998) concept of primary emotion circuits. It consists of 110 items rated on a 4-point Likert scale ranging from 1 (“strongly disagree”) to 4 (“strongly agree”). The scales of the ANPS include “SEEKING,” “SADNESS,” “FEAR,” “ANGER,” “CARE,” and “PLAY” as well as an additional scale for spirituality.

The *Adult Attachment Scale* (AAS; Collins and Read, 1990; German version: Schmidt et al., 2004), which assesses the subject’s anxiety about being rejected or unloved (“Anxiety”); comfort with closeness (“Close”) and comfort with depending on others (“Depend”). The German version consists of 15 items (five items per scale), which are rated on a 5-point Likert scale ranging from 1 (“strongly disagree”) to 5 (“strongly agree”).

The *Operationalized Psychodynamic Diagnostics Structure Questionnaire* (OPD-SQS; Ehrenthal et al., 2015) is a self-report measurement, which assesses deficits in personality functioning as proposed in the Operationalized Psychodynamic Diagnostic (Arbeitskreis OPD-2, 2008). This measure is comprised of 91 items which are rated on a 5-point Likert scale ranging from 0 (“strongly disagree”) to 4 (“strongly agree”). The total score indicates deficits in overall personality functioning, with higher scores indicating more severe impairments.

The *Brief Symptom Inventory* (BSI-18; Derogatis, 2001; German version: Spitzer et al., 2011) is comprised of 18 items which measure the amount of symptom burden in the last 7 days with regards to depression, anxiety and somatization. Items are rated on a 5-point Likert scale ranging from 0 “absolutely not” to 4 “very strong.” The total score “Global Severity Index” can be generated by adding the scores of every item.

### Statistical Analysis and Analysis Strategy

The confirmatory factor analysis (CFA) was conducted with AMOS 26. SPSS 27.0 was used for data management, descriptive statistics and bivariate correlations. Goodness-of-fit was assessed with maximum likelihood (ML) estimation in AMOS. In accordance with Kline (2015), the following global fit-indices were considered as markers for an acceptable model fit: (a) The Comparative Fit Index (*CFI*) >0.90; (b) Tucker-Lewis Index (*TLI*) >0.90; (c) the Normed Fit Index (NFI) >0.90; (d) the Relative Fit Index (RFI) >0.90; (e) the square root error of approximation (*RMSEA*) <0.08 and the upper bound of its 90% confidence interval <1; and (f) χ^2^/df < 3.

For the comparison of competing models, the Akaike Information Criterion (*AIC*) was used, which rewards models that achieve a high goodness-of-fit and penalizes them if they become overly complex (Kline, 2015). In this context, the model with the smallest *AIC* value was preferred, with a Δ*AIC* > 2 indicating significant differences (Cheung and Rensvold, 2002; Jovanović, 2015).

To establish model identification one factor loading was fixed to 1 for each factor in every specified model (Byrne, 2004).

## Results

### Sample Characteristics and Descriptive Statistics

The investigated sample consisted of 371 German-speaking adults (58.50% female) which were aged 18–69 years (*M* = 28; *SD* = 9.75). Most participants (39%) had a general qualification for university entrance as highest finished education, were in education (53%) and Austrian (80%). Table 1 displays the sample characteristics in detail. Item characteristics of L-12 and L-5 are shown in Table 2. Item-total correlation ranged from *r*_*iT*_ = 0.54–0.68 for both scale versions. Item difficulty ranged from 45.22 to 63.07 in L-12 and from 45.22 to 57.01 in L-5.

**TABLE 1:** Sample characteristics (*N* = 371).

Sample	*N* or *M*	% or SD
Gender	*N* = 217 Female/	58.50%
	*N* = 154 Male	41.50%
Age	*M* = 28	*SD* = 9.75 years
Highest finished education	*N* = 30 High School/	8.10%
	*N* = 146 General qualification for university entrance/	39.35%
	*N* = 72 Bachelor University degree/	19.41%
	*N* = 68 Master University degree/	18.32%
	*N* = 44 Apprenticeship/	11.86%
	*N* = 11 Ph.D./	2.96%
Occupation	*N* = 150 In employment/	40.43%
	*N* = 198 In education/	53.34%
	*N* = 18 Unemployed/	4.85%
	*N* = 5 in Pension	1.35%
Relationship Status	*N* = 45 Married/	12.12%
	*N* = 162 In Relationship/	43.67%
	*N* = 164 Single/	44.20%
Nationality	*N* = 291 Austrian/	80.05%
	*N* = 79 German/	21.29%
	*N* = 11 Other	2.96%

**TABLE 2:** Item-characteristics of L-12 and L-5 scales.

Scale and Items	X̄_i_	S_i_	r_iT_	P_i_
**L-12**				
1. Mir fällt es leicht, mich erotischen Erfahrungen hinzugeben	2.81	0.89	0.63	45.22
2. Meine Sexualität auszuleben, fühlt sich irgendwie nicht richtig an*	3.27	0.87	0.57	56.67
3. Ich empfinde meine Sexualität allgemein als befriedigend	2.93	0.86	0.62	48.32
4. Sexualität ist für mich mit Ekel verknüpft*	3.52	0.77	0.65	63.07
5. Mir fällt es leicht, einen Orgasmus zu haben	2.95	0.89	0.53	48.72
6. Ich stehe Sexualität nicht besonders offen gegenüber*	3.19	0.88	0.63	54.78
7. Ich kann das Ausüben von sexuellen Handlungen (Geschlechtsverkehr, Masturbation, etc.) voll und ganz genießen	3.25	0.79	0.73	56.20
8. Mit meiner Sexualität habe ich häufig schlechte Erfahrungen gemacht*	3.28	0.84	0.58	57.01
9. Wenn ich Sex habe, komme ich meist zu einem Orgasmus	3.04	0.99	0.57	50.94
10. Den Anblick von Geschlechtsorganen finde ich abstoßend*	3.42	0.80	0.60	60.51
11. Wenn ich einen Orgasmus habe, nehme ich ihn meistens sehr intensiv wahr	2.99	0.84	0.54	49.87
12. Sexualität ist für mich sehr mit Scham besetzt*	3.22	0.89	0.68	55.59
**L-5**				
1. Mir fällt es leicht, mich erotischen Erfahrungen hinzugeben	2.81	0.89	0.66	45.22
2. Mit meiner Sexualität habe ich häufig schlechte Erfahrungen gemacht*	3.28	0.84	0.54	57.01
3. Ich empfinde meine Sexualität allgemein als befriedigend	2.93	0.86	0.64	48.32
4. Ich stehe Sexualität nicht besonders offen gegenüber*	3.19	0.88	0.54	54.78
5. Ich kann das Ausüben von sexuellen Handlungen (Geschlechtsverkehr, Masturbation, etc.) voll und ganz genießen	3.25	0.79	0.68	56.20

N = 371; ${\bar{\text{X}}}_{i}$, Mean of Item scores; s_i_, Standard Deviation of Item scores; r_iT_, Item Total Correlation; P_i_, Item Difficulty. *Items are scored inversely.

With regard to sex differences, results indicated higher L-12 scores in males [*F*_(1_,_369)_ = 6.49; *p* < 0.05; *M*_*male*_ = 3.25; *SD*_*male*_ = 0.56; *M*_*female*_ = 3.09; *SD*_*female*_ = 0.60] with a small effect size of η^2^ = 0.02, and no differences in L-5 [*F*_(1_,_369)_ = 0.71; *p* > 0.05].

### Normal Distribution

Inspection of skewness and kurtosis indicated a normal distribution of both L-12 (skewness = 0.82; kurtosis = 0.74) and L-5 (skewness = −0.67; kurtosis = 0.22).

### External Validity

L-12 and L-5 exhibited a large correlation (*r* = 0.92; *p* < 0.001; see Cohen, 1992). Consequently, both scales showed a similar correlation pattern with external variables (see Table 3). While neither exhibited a significant relationship with ANGER and Spirituality (both *p* > 0.05), small to moderate positive correlations were observed with SEEKING, PLAY, Trust, and Closeness (*r* = 0.28–0.35; all *p* < 0.001). In contrast, L-12 and L-5 exhibited small to moderate negative correlations with SADNESS, and anxiety about being rejected or unloved, personality functioning deficits, and psychiatric symptom burden (*r* = −0.25 to −0.39; all *p* < 0.001). CARE showed a small positive correlation with L-5 (*r* = 0.14; *p* < 0.01) but not L-12 (*p* > 0.05).

**TABLE 3:** Descriptive statistics, internal consistency and correlations between the L-scales and measures of personality structure as well as psychiatric symptom burden.

Measurement		Variable	1	2	3	4	5	6	7	8	9	10	11	12	13	14	15	16	17
LUST	1.	L-12	–																
	2.	L-5	0.93**	–															
ANPS	3.	SEEKING	0.30**	0.32**	–														
	4.	FEAR	−0.30**	−0.31**	−0.24**	–													
	5.	CARE	0.13	0.14*	0.24**	0.14*	–												
	6.	PLAY	0.32**	0.35**	0.50**	−0.30**	0.39**	–											
	7.	ANGER	–0.04	–0.06	–0.02	0.33**	–0.03	–0.11	–										
	8.	SADNESS	−0.25**	−0.27**	−0.25**	0.68	0.08	−0.29**	0.37**	–									
	9.	Spirituality	0.05	0.05	0.13	–0.06	0.13	0.15*	–0.06	0.01	–								
BSI-18	10.	Somatization	−0.21**	−0.21**	–0.10	0.31**	0.03	−0.17**	0.23**	–0.03	0.30**	–							
	11.	Depression	−0.29**	−0.33**	−0.30**	0.50**	–0.07	−0.34**	0.21**	–0.03	0.59**	0.48**	–						
	12.	Anxiety	−0.20**	−0.22**	–0.10	0.48**	0.03	−0.17**	0.27**	–0.01	0.45**	0.63**	0.63**	–					
	13.	GSI	−0.28**	−0.31**	−0.21**	0.52**	–0.01	−0.28**	0.28**	–0.03	0.55**	0.79**	0.87**	0.88**	–				
OPD	14.	PS	−0.37**	−0.39**	−0.28**	0.55**	–0.10	−0.37**	0.25**	0.09	0.54**	0.52**	0.63**	0.58**	0.69**	–			
AAS	15.	Trust	0.28**	0.29**	0.25**	−0.31**	0.21**	0.45**	−0.29**	0.07	−0.40**	−0.36**	−0.54**	−0.35**	−0.51**	−0.54**	–		
	16.	Closeness	0.37**	0.38**	0.28**	−0.26**	0.25**	0.50**	–0.14	0.11	−0.25**	−0.33**	−0.38**	−0.30**	−0.40**	−0.56**	0.52**	–	
	17.	Anxiety	−0.27**	−0.28**	−0.19**	0.53**	0.09	−0.20**	0.22**	0.00	0.64**	0.34**	0.55**	0.52**	0.57**	0.66**	−0.44**	−0.27**	–
Descriptive		*M*	3.16	3.09	2.89	2.64	2.90	2.90	2.53	2.30	2.52	9.57	11.19	1.71	31.48	64.43	15.94	13.24	11.06
		SD	0.59	0.65	0.37	0.52	0.41	0.45	0.48	0.62	0.41	3.79	5.37	4.00	11.17	18.37	4.57	4.75	4.43
		α	0.90	0.82	0.74	0.87	0.75	0.82	0.84	0.87	0.74	78	0.88	0.80	0.91	0.96	0.82	0.86	0.77

N = 371; ANPS, Affective Neuroscience Personality Scales; GSI, BSI-18 Total Score; PS, OPD Personality Structure; M, mean; SD, standard deviation α, Cronbach’s α. *p < 0.01; **p < 0.001.

### Reliability

Cronbach’s α indicated excellent internal consistency regarding L-12 (α = 0.90), while L-5 showed acceptable reliability (α = 0.82).

### Confirmatory Factor Analyses of the LUST-Scale

Confirmatory factor analysis fit indices for the 12-item and 5-item version are detailed in Table 4. An initial solution of the 12-item version without correlated error terms estimated a poor fitting model: χ*^2^/*df = 8.02; *RMSEA* = 0.14 (90% CI: 0.13, 0.15); *CFI* = 0.81; *NFI* = 0.79; *TLI* = 0.77; *RFI* = 0.74.

**TABLE 4:** Results of the confirmatory factor analysis.

Model	χ^2^ (df)	χ^2^/df	RMSEA (90% CI)	CFI	NFI	TLI	RFI
12-Item version (without error term correlation)	433.13 (54)	8.02	0.138 (0.128–0.150)	0.810	0.790	0.768	0.744
12-Item version (Bifactor model)	84.22 (42)	2.01	0.053 (0.036–0.069)	0.979	0.959	0.967	0.936
5-Item version	2.79 (5)	0.56	0.000 (0.000–0.052)	1.000	0.995	1.007	0.991

*N = 371.*

Based on the inspection of error term correlations, a bifactor model of the L-12 scale was investigated, which specified a general factor (L-12) and two residual factors modeled via the assignments of (1) positively poled items and (2) negatively poled items. Results indicated an excellent model fit: χ^2^/df = 2.02; *RMSEA* = 0.05 (90% CI: 0.04, 0.70); *CFI* = 0.97; *NFI* = 0.96; *TLI* = 0.97; *RFI* = 0.93.

L-5 showed an excellent fit with the following indices: χ*^2^/*df = 0.56; *RMSEA* = 0.00 (90% CI: 0.00, 0.05); *CFI* = 1.00; *NFI* = 1.00; *TLI* = 1.01; *RFI* = 0.99.

**FIGURE 2:**
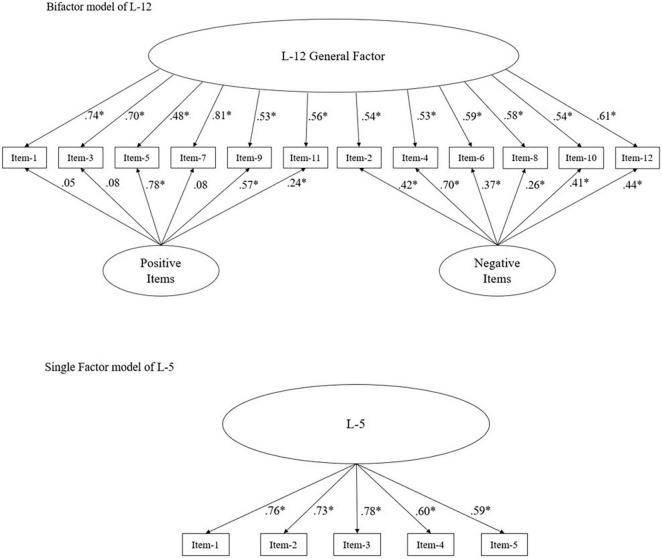
Confirmatory factor analysis of L-12 [χ^2^/df = 2.02; RMSEA = 0.05 (90% CI: 0.04, 0.70); CFI = 0.97; NFI = 0.96; TLI = 0.97; RFI = 0.93] and L-5 [χ^2^/df = 0.56; RMSEA = 0.00 (90% CI: 0.00, 0.05); CFI = 1.00; NFI = 1.00; TLI = 1.01; RFI = 0.99]; *N* = 371; Items with even numbers are scored inversely. ^∗^*p* < 0.001.

As detailed in Figure 2, the factor loadings of the initial L-12 model ranged from β = 0.53 to β = 0.73 (all *p* < 0.001). Regarding the bifactor model all associations with the total factor ranged from β = 0.53 to β = 0.81, while significant associations with the positive item residual factor were estimated between β = 0.24 and β = 0.78 (all *p* < 0.001) and between β = 0.26 to β = 0.70 for the negative item residual factor (all *p* < 0.001). Three items did not show significant association with either residual factor (all *p* > 0.05).

Results for the five-item version indicated factor loadings ranging from β = 0.53 to β = 0.76 (all *p* < 0.001).

## Discussion

This study aimed to be a building block toward the development of a self-rate measurement for the operationalization of LUST as proposed by Panksepp (1998). The psychometric properties presented in this paper suggest high reliabilities, normal distribution, good structural validity and a nomological network conforming to our hypotheses of both the long- and short-version of the proposed L-scales.

With respect to reliability, L-12 and L-5 exhibited excellent and good internal consistency, respectively, with comparable or even higher Cronbach’s α than the scales of the ANPS. For future studies further markers of reliability (e.g., split half or retest reliability) should be estimated. Additionally, inspection of skewness and kurtosis suggested a normal distribution of both scales with regard to a healthy population, again resonating with the characteristics of original ANPS (Davis et al., 2003; Davis and Panksepp, 2011).

Confirmatory factor analysis of L-12 revealed a bifactorial structure, with one general latent factor and two residual factors which seem to reflect specific variance regarding different answering styles with respect to items formulated positively vs. items which assessed LUST inversely. Alternatively, this result might be interpreted as residual variance caused by items concerning orgasms and items operationalizing disgust and shame, respectively. However, this effect vanished in the short version. L-5 exhibited excellent model fit in a single factor model, making it especially useful for studies applying structural equation modeling.

As the original version of the ANPS is often considered too long, currently two brief versions of the questionnaire have been developed, namely the ANPS short (ANPS-S, Pingault et al., 2012), and the brief ANPS (BANPS, Barrett et al., 2013). According to Pedersen et al. (2014) short versions generally improve the psychometric properties of the ANPS, however, the authors abstained from constructing a corresponding LUST scales. In this context, the L-5 scale might prove itself as a practical supplement for both ANPS short versions. In correspondence to this, the present form of the L-Scales was constructed in accordance with 5-point response format of the BANPS and consequently differs from 4-point format of the original ANPS.

The construction of the L-scales was aimed to be closely aligned to the original definition of LUST as introduced by Panksepp (1998, 2012), hence the investigated items consist of statements associated with eroticism, sexual enjoyment and pleasure. Of note, for the short version of the questionnaire, all statements which mentioned orgasms had to be deleted in order to establish a robust single-factor structure. Considering significant sex differences in the frequency of orgasms during intercourse (Frederick et al., 2018), the exclusion of statements focused on orgasms might explain the disappearance of sex differences in the short version. Nevertheless, both L-12 and L-5 exhibit a strong intercorrelation and a very similar correlation pattern with external criteria.

In correspondence to this, the L-scales exhibited moderate positive correlations with dispositions toward SEEKING and PLAY, while there were only small to non-significant correlations with CARE. Furthermore, both scales showed moderate negative correlations with SADNESS and FEAR, whereas both were independent of ANGER. Accordingly, the L-scales appear to be more inversely related to negative primary emotions than the scale developed by van der Westhuizen and Solms (2015). Hence, the results resonate with current neuropsychoanalytic literature, which assumes underlying drives (libidinal vs. destructive) acting as a latent grouping mechanism for affects (Krause, 2012; Kernberg, 2015; Boeker et al., 2018). However, further studies employing structural equation modeling which consider all seven primary emotions in a single model should be done to further investigate this hypothesis.

In accordance with the current psychodynamic understanding of the functional role of attachment and personality structure regarding affect regulation (Krause, 2012; Kernberg, 2015; Fonagy, 2018), the observed positive correlations between LUST and attachment security as well as more mature personality structure echo recent evidence indicating secure attachment and personality functioning to predict a pattern of increased positive and decreased negative primary emotions (Fuchshuber et al., 2019).

Along these lines, LUST appeared to be negatively related to increased psychiatric symptom burden. More detailed inspection suggests that this is predominantly driven by its negative association with depressive symptoms. Nevertheless, we also found significant (but small) negative correlations with symptoms of anxiety and somatization. These findings are in agreement with the current review of Rokach (2019), who detected that sexual dysfunctions are a wide spread concomitant of affective disorders. With regard to clinically relevant associations proposed by Panksepp (2004) future research should investigate the relationship between the L-scales and pathological jealousy and hypersexuality.

What is more, neither scale showed a significant correlation with spirituality as assessed with the ANPS. Relatively little is known about the relationship between LUST and spirituality, however, Brelsford et al. (2011) reported findings indicating a moderate negative correlation of sexuality and spirituality. Nevertheless, considering spiritual traditions which involve sexual practices (e.g., Tantra) further research may be needed to evaluate this relationship in more detail.

### Limitations and Future Perspectives

Several limitations of this study must be noted which restrain the interpretation of the results and need to be addressed in future research.

A next step in the validation of the L-scales will be the evaluation of its external validity based on measures of sexuality like the Sexual Desire Inventory (SDI, Spector et al., 1996) or the Multidimensional Sexuality Questionnaire (Snell et al., 1993). While further convergent validity tests are needed, the L-scales presented here have high face validity (i.e., item content clearly assess the individual capacity to attain sexual pleasure), and results provide clear evidence for a common factor underlying all items, as well as a nomological network with respect to relevant aspects of personality which is in good accordance with our hypotheses.

Another critical issue of the current L-scales is that neither the long- nor the short-version take into account the frequency of sexual behavior as well as the aspect of sexual desire, as corresponding items were excluded based on initial item statistics and considerations regarding internal consistency. Nevertheless, sexual urges and the prevalence of sexual behavior are usually (but not always; see e.g., Solms and Turnbull, 2002) proposed as important markers for the expression of LUST (Panksepp and Biven, 2012). Therefore, a revised version of the L-scales should aim at reformulation and inclusion of associated items.

The present study is further limited due to the nature of its sample. The convenient sample was recruited online – an approach which is often controversially discussed in literature (see e.g., Wiersma, 2013) – and predominantly consisted of healthy students from Austrian universities. Thereby, the presence of psychiatric disorders was assessed via self-report and hence, there is a lack of systematic assessment by eligible diagnostic instruments (e.g., SKID, First et al., 2002). Along these lines, the L-scales needs to be evaluated in clinical populations in order to learn more about its etiological relevance.

Finally, our findings are restricted due to a lack of differentiation regarding the sexual orientation or gender identity of participants, as no plausible *a priori* reasons of significant differences were assumed.

### Conclusion

This study aimed to complement the ANPS framework by a LUST scale – a concept which has been discussed as a vital element yet lacks a standardized assessment. The present data suggests that the L-scales scales are distinguished by high reliability, satisfying structural validity and plausible correlations with external criteria. Therefore, this study might serve as vital groundwork for a standardized operationalization of LUST. However, more research will be necessary to further evaluate this questionnaire, especially with considerations to external validity and its applicability in clinical populations.

## Data Availability Statement

The raw data supporting the conclusions of this article will be made available by the authors, without undue reservation.

## Ethics Statement

The studies involving human participants were reviewed and approved by the Medical University of Graz. The patients/participants provided their written informed consent to participate in this study.

## Author Contributions

JF conducted the all statistical analysis and wrote the first draft of the manuscript. EJ, MH-R, and HU read the manuscript and made some critical comments. JF, MH-R, EJ, and HU revised the whole manuscript. All authors contributed to the article and approved the submitted version.

## Conflict of Interest

The authors declare that the research was conducted in the absence of any commercial or financial relationships that could be construed as a potential conflict of interest.

## Publisher’s Note

All claims expressed in this article are solely those of the authors and do not necessarily represent those of their affiliated organizations, or those of the publisher, the editors and the reviewers. Any product that may be evaluated in this article, or claim that may be made by its manufacturer, is not guaranteed or endorsed by the publisher.
