# Supplementary material for: The Affective Neuroscience of Sexuality: Development of a LUST Scale
Source: Front Hum Neurosci. 2022 Feb 28;16:853706. doi: 10.3389/fnhum.2022.853706 (PMC8918489; doi:10.3389/fnhum.2022.853706)
Supplement: Supplementary file 1 [file Table_1.DOCX]

| **English Translation of L-12 and L-5** |
| --- |
| **L-12** |
| 1. For me, it is easy to indulge myself in erotic experiences. |
| 2. Acting out my sexuality somehow doesn't feel right. * |
| 3. I am generally satisfied with my sexuality. |
| 4. For me, sexuality is associated with disgust. * |
| 5. For me, it is easy to have an orgasm. |
| 6. I'm not particularly open about sexuality. * |
| 7. I can fully enjoy engaging in sexual activities (intercourse, masturbation, etc.). |
| 8. I have often had negative experiences with my sexuality. * |
| 9. When I have sex, I usually have an orgasm. |
| 10. I find the sight of genitals repulsive. * |
| 11. When I have an orgasm, I usually experience it very intensely. |
| 12. For me, sexuality is very much associated with shame. * |
| **L-5** |
| 1. For me, it is easy to indulge myself in erotic experiences. |
| 2. I have often had negative experiences with my sexuality. * |
| 3. I am generally satisfied with my sexuality. |
| 4. I'm not particularly open about sexuality. * |
| 5. I can fully enjoy engaging in sexual activities (intercourse, masturbation, etc.). |
